# Molecular Mechanisms Underlying Synaptic and Axon Degeneration in Parkinson’s Disease

**DOI:** 10.3389/fncel.2021.626128

**Published:** 2021-03-02

**Authors:** Nolwazi Z. Gcwensa, Drèson L. Russell, Rita M. Cowell, Laura A. Volpicelli-Daley

**Affiliations:** ^1^Department of Neurobiology, Center for Neurodegeneration and Experimental Therapeutics, Civitan International Research Center, Birmingham, AL, United States; ^2^Department of Neuroscience, Southern Research, Birmingham, AL, United States

**Keywords:** α-synuclein, synapse, Parkinson’s disease, Dementia with Lewy Bodies, GWAS, degeneration

## Abstract

Parkinson’s disease (PD) is a progressive neurodegenerative disease that impairs movement as well as causing multiple other symptoms such as autonomic dysfunction, rapid eye movement (REM) sleep behavior disorder, hyposmia, and cognitive changes. Loss of dopamine neurons in the substantia nigra pars compacta (SNc) and loss of dopamine terminals in the striatum contribute to characteristic motor features. Although therapies ease the symptoms of PD, there are no treatments to slow its progression. Accumulating evidence suggests that synaptic impairments and axonal degeneration precede neuronal cell body loss. Early synaptic changes may be a target to prevent disease onset and slow progression. Imaging of PD patients with radioligands, post-mortem pathologic studies in sporadic PD patients, and animal models of PD demonstrate abnormalities in presynaptic terminals as well as postsynaptic dendritic spines. Dopaminergic and excitatory synapses are substantially reduced in PD, and whether other neuronal subtypes show synaptic defects remains relatively unexplored. Genetic studies implicate several genes that play a role at the synapse, providing additional support for synaptic dysfunction in PD. In this review article we: (1) provide evidence for synaptic defects occurring in PD before neuron death; (2) describe the main genes implicated in PD that could contribute to synapse dysfunction; and (3) show correlations between the expression of *Snca* mRNA and mouse homologs of PD GWAS genes demonstrating selective enrichment of *Snca* and synaptic genes in dopaminergic, excitatory and cholinergic neurons. Altogether, these findings highlight the need for novel therapeutics targeting the synapse and suggest that future studies should explore the roles for PD-implicated genes across multiple neuron types and circuits.

## Introduction

### An Overview of Parkinson’s Disease

Parkinson’s disease (PD) is a neurodegenerative disease that typically occurs in individuals older than 65 with symptoms of impaired motor function including muscular rigidity, slowness of movement (bradykinesia), impaired voluntary movement (akinesia), abnormalities in posture and gait, and resting tremor. Non-motor symptoms can also develop such as olfactory dysfunction (hyposmia), cognitive impairment, autonomic dysfunction, and REM sleep behavior disorder.

Pathologically, PD is characterized by the loss of dopamine neurons in the substantia nigra pars compacta (SNc). SNc dopamine neurons project poorly myelinated axons rostrally to the caudate and putamen in the human brain (striatum in rodents) where dopamine release plays an important role in regulating the neuronal activity of striatal spiny projection neurons (SPNs). SPNs send axonal projections to either the direct or indirect basal ganglia pathways composed of multiple brain regions: the globus pallidus internal (GPi), globus pallidus external (GPe), substantia nigra pars reticulata (SNr), subthalamic nucleus, thalamus, and cortex (Calabresi et al., 2014). Loss of dopamine in the striatum ultimately results in inhibition of the thalamus and reduced cortical activity, producing motor symptoms such as rigidity and bradykinesia. It should be noted that the basal ganglia circuitry and altered patterns on neuronal firing causing symptoms of PD are not nearly as simplistic as stated above and outstanding research on changes in firing patterns within basal ganglia circuitry is advancing the field (Wichmann, 2019).

Loss of dopamine neurons in the SNc in PD is well-established but, it is important to point out that neuron loss occurs elsewhere in the brain. Rigorous evaluation of cell loss in PD confirms degeneration in the SNc, pedunculopontine nucleus, supraoptic nucleus, amygdala, the center median-parafascicular region of the thalamus, and the pre-supplementary motor cortex (Surmeier et al., 2017). A recent review on cell loss throughout the PD brain took great care to analyze all the studies (ranging from 1953 to 2015) demonstrating cell loss and classified whether these studies utilized unbiased stereology to count neuron populations within brain nuclei (Giguere et al., 2018). Stereological methods are the most precise method of analyzing neuron loss because of the rigorous sampling methods, avoidance of counting the same neuron more than once, and stereology accounts for differences in tissue thickness. In the pedunculopontine nuclei, significant loss of cholinergic neurons was reported in 11 studies with four of those using unbiased stereology and counting both cholinesterase positive neurons and generic neuron markers to ensure cell loss and not just loss of neuron phenotype. Loss of cholinergic neurons has also been reported in the dorsal motor nucleus of the vagus (one study using stereology). Loss of noradrenergic neurons has been reported in 18 studies but none of these studies utilized unbiased stereology. Similarly, while the loss of neurons in the raphe nucleus has been reported, the one study using unbiased stereology reported no neuron loss in PD. Surprisingly, although the ventral tegmental nucleus is assumed to be unaffected in PD, loss of neurons in this brain region has been reported in eight studies including one using unbiased stereology. In the amygdala, one stereology study showed a 30% loss of neurons (Harding et al., 2002). Overall, clearly, there is cell loss in brain areas in addition to the SNc, but neuron loss in these other brain areas requires further validation. It is also important to consider that while the cells might not die in PD, they could be but how these other neurons and circuits contribute to PD symptoms, and in which circuits synaptic dysfunction occurs remains unclear.

Most cases of sporadic late-onset PD are also characterized by proteinaceous inclusions composed mostly of the protein α-synuclein found in the neuronal cell body, called Lewy bodies (LB), and axons, and dendrites, called Lewy neurites (LN). Staging studies using postmortem brains from individuals ranging from early stages of PD to advanced stages show that Lewy pathology first appears in the olfactory bulb, the enteric nervous system of the gut, and the dorsal motor nucleus of the vagus located in the medulla oblongata. Lewy pathology then appears in locus coeruleus in the pons, the raphe nucleus in the pons-midbrain, and the SNc in the midbrain. At the late stages of the disease, Lewy pathology is found in the temporal cortex, limbic regions, and cortex.

It is worthwhile to mention another neuronal synucleinopathy, Dementia with Lewy Bodies (DLB) which shares similarities to PD. This disease is characterized by cognitive symptoms, hallucinations, fluctuations in attention, and motor symptoms similar to PD; cognitive symptoms appear first followed by motor symptoms a year or more later. Similar to PD, DLB is also characterized by loss of dopamine terminals in the striatum and dopamine neurons in the SNc. In DLB, Lewy pathology is more abundant in limbic and cortical regions than in PD (Beach et al., 2009), although Lewy pathology in DLB is more abundant overall than PD in most brain regions analyzed. Interestingly, patients with rare dominantly inherited mutations in α-synuclein, or α-synuclein triplications, present with severe cognitive and psychiatric symptoms (Polymeropoulos et al., 1997; Spira et al., 2001; Singleton et al., 2003; Kiely et al., 2013; Lesage et al., 2013) and maybe more accurately described as DLB patients rather than PD. Regardless, studies of DLB can help researchers understand the consequences of abnormal α-synuclein on synapse structure and function.

Presently, treatments for PD successfully target the associated motor symptomology. Levodopa (L-dopa), a precursor for dopamine synthesis, alleviates bradykinesia and rigidity with mixed efficacy concerning tremor and posture and gait problems (Connolly and Lang, 2014). Over time, L-dopa shows fluctuations in efficacy with reduced “on” times when the medication alleviates motor symptoms, and more frequent “off” times when motor symptoms return. The diminished efficacy of L-dopa likely relates to the progressive loss of dopamine neurotransmission. Also, while L-dopa is very effective in alleviating motor symptoms, it does not treat the non-motor symptoms of PD including cognitive dysfunction or autonomic impairments. In a study in which patients received L-dopa or placebo for 42 weeks followed by a 2-week wash-out in which patients received no drug, the patients taking L-dopa did not show the same extent of deterioration of symptoms compared to the placebo group. These data suggest that L-dopa may produce a long-lasting beneficial effect on symptom progression, which is likely mediated by the plasticity of corticostriatal and nigrostriatal signaling in the striatum (Surmeier et al., 2007; Calabresi et al., 2015; Albin and Leventhal, 2017; Zhai et al., 2019). However, L-dopa does not prevent the inevitable death of dopamine neurons, nor the continual formation of Lewy pathology throughout the brain (Olanow, 2019).

By the time motor symptoms are diagnosed, PD is far advanced with 50–60% loss of dopamine neurons in the SNc and up to 80% loss of terminals, particularly in the putamen (Kordower et al., 2013; Kurowska et al., 2016). The prodromal phase before PD diagnosis can last anywhere from 5–20 years and represents a stage in which interventions could prevent disease progression. Imaging of dopaminergic terminals in the putamen using single-photon emission computed tomography (SPECT) and ligands to the dopamine transporter (DAT), suggests significant striatal terminal loss during this prodromal stage(Jennings et al., 2017; Fazio et al., 2018; Delva et al., 2020). Pathological studies in post-mortem brains demonstrate that loss of dopaminergic terminals and axons in the putamen occur in the early stages of PD before the loss of dopamine neurons (Kordower et al., 2013). Loss of glutamatergic synapses in the striatum, as well as other synapses, also occurs in early PD (McNeill et al., 1988; Anglade et al., 1996; Zaja-Milatovic et al., 2005; Villalba and Smith, 2018; Delva et al., 2020; Matuskey et al., 2020) although the extent of loss varies depending on the region of the striatum analyzed (caudate vs. putamen, patches vs. matrix, direct vs indirect pathway), as well as disease stage and medication status. Understanding the mechanisms of synapse alterations in PD may help us to find treatments that protect against synapse dysfunction and loss, or that restore synapses before intractable neuron death, thus preventing PD progression.

## Imaging Studies Demonstrating Synaptic Loss in Individuals Living with PD

Although much of the focus of the PD field has been on the death of dopamine neurons in the SNc, it has been hypothesized PD initiates with loss of dopamine terminals and axons in the striatum and that this should be the target of therapeutics (Chu et al., 2012; Kordower et al., 2013; Kurowska et al., 2016; Kordower and Burke, 2018; Wong et al., 2019). This inference was based on discoveries around the reduced localization of DATs enriched in the striatal dopamine terminals. Accumulating evidence supports that loss of presynaptic terminals initiates PD and that there is a greater loss of striatal terminals relative to the loss of dopaminergic soma in the SNc. For example, positron emission tomography (PET) imaging using a radioligand DAT, (^123^I)β-CIT SPECT DAT, showed that of individuals with both hyposmia and low baseline levels of DAT, 67% converted to PD over 4 years (Jennings et al., 2017). The risk of conversion to PD in individuals with hyposmia and an initial DAT deficit was 17.47 (95% CI, 7.02–43.45). High resolution, selective imaging of dopaminergic presynaptic terminals in human brains has recently been facilitated by the development of [^18^F]-(E)-N-(3-iodoprop-2-enyl)-2β-carbofluoroethoxy-3β-(4′-methyl-phenyl) nortropane (^18^F-FE-PE2I). In early PD patients, ^18^F-FE-PE2I shows an approximately 75.6% loss of dopamine terminals in the putamen compared to healthy controls (Fazio et al., 2018; Delva et al., 2020).

Human studies also indicate degeneration of other synapses in addition to dopamine terminals. For example, two recent imaging studies using (R)-1-([3-(^11^C-methyl-^11^C)pyridin-4-yl)methyl]-4-(3,4,5-trifluorophenyl)pyrrolidine-2-one (^11^C-UCB-J), a PET radioligand for the synaptic vesicle protein 2A (SV2A), a marker for most synaptic terminals in the brain, shows loss of synaptic terminals in the SNc. This suggests presynaptic abnormalities possibly arising from projection neuron axons from the striatum, globus pallidus, subthalamic nucleus, pedunculopontine nucleus, and/or the amygdala, brain regions that all project to the SNc (Delva et al., 2020; Matuskey et al., 2020). High resolution, quantitative imaging of post-mortem tissue would help confirm these imaging results. In the future, the development of imaging agents to SV2C, which is restricted to brain areas vulnerable in PD such as the globus pallidus, SNc, and olfactory bulb, may find more robust and significant loss that corresponds to the development of symptoms (Janz and Südhof, 1999).

It is important to note that imaging studies are limited because only one synaptic marker can be analyzed at one time. Future confirmation of the imaging findings will be important for distinguishing phenotypic loss of DAT or synaptic vesicle markers as opposed to the loss of terminals and axons. For example, impaired anterograde axonal transport of DAT in PD could cause reduced binding of PET ligands for this transporter, while the terminals themselves remain. Also, impairments in the recycling of synaptic vesicles could result in less SV2A binding without loss of synapses. If impaired trafficking instead of terminal loss is discovered, these could still have important implications for the PD field. Future pathological studies using multiple synaptic markers as well as DAT will be useful, as well as animal models of PD which allow for better spatial and temporal microscopic resolution.

## Pathological Studies Demonstrating Reduced Dopamine Terminal and Axon Markers in Human PD Striatum

Postmortem studies have confirmed the progressive loss of DA terminals with disease onset. Immunostaining was used to track nigrostriatal degeneration in brain sections of PD sufferers at different intervals post-diagnosis. Using quantitative immunohistochemistry to TH and DAT, PD brains, relative to controls, showed 35–75% loss of dopamine terminals in the putamen at years 1–3 after diagnosis, and 70–90% loss by year 5 after diagnosis relative to controls. In the SNc, PD brains showed 50–90% loss of TH-positive cell bodies at years 1–3 after diagnosis with minimal further loss over several years after diagnosis. This study suggests that progressive loss of dopamine terminals in the putamen may coincide better with progressive worsening of symptoms compared to the loss of dopamine neurons in the SNc (Kordower et al., 2013). In addition to the loss of nigrostriatal axons, loss of dopamine projections from the SNc to the amygdala has been reported and may contribute to psychosis and anxiety in PD dementia and DLB (Iseki et al., 2001).

The findings of terminal loss were recently replicated in a rat model of PD in which α-synuclein fibrils were injected into the striatum which induces endogenous α-synuclein to form inclusions resembling those found in PD brains. PET imaging of the rats using [^11^C]DTBZ, a marker of vesicular monoamine-2 transporter demonstrated loss of striatal dopamine terminals 6 weeks after fibril injection, a time point at which there was no loss of TH-positive dopamine neurons in the SNc (Thomsen et al., 2020). These results were confirmed with immunohistochemistry in the striatum to TH, DAT, and VMAT2. The rat or mouse fibril model could be used in the future to determine the course of terminal loss in the striatum that occurs relative to α-synuclein aggregation and to possibly discover novel methods of preventing terminal loss.

Although most of the PD field has focused on nigrostriatal neurons in the SNc, glutamatergic synapses in the striatum are also altered in PD. The striatum is densely innervated with glutamatergic terminals originating from the thalamus or cortex that synapse on GABAergic spiny projection neurons (SPN). The glutamatergic synapses occur on dendritic spines of SPNs. Cortico-striatal and thalamo-striatal glutamatergic synaptic transmission in the striatum is, in turn, facilitated by dopamine from the SNc. Postmortem studies of brains from advanced PD patients show a robust loss in spine density in spiny projection neurons, suggesting loss of excitatory corticostriatal and thalamostriatal glutamatergic synapses in the striatum (McNeill et al., 1988; Anglade et al., 1996; Stephens et al., 2005; Zaja-Milatovic et al., 2005; Villalba and Smith, 2018).

Reduced levels of dopamine contribute to the loss of glutamatergic synapses and SPN spines (Day et al., 2006; Graves and Surmeier, 2019), even in the earlier stages of the disease with partial loss of dopamine in the striatum (Paillé et al., 2010). Alterations in glutamatergic synaptic transmission is initially a homeostatic mechanism to maintain activity in the basal ganglia. However, over time, loss of glutamatergic SPN synapses causes defects in network activity. It has also been hypothesized that loss of dopamine axons may be caused by abnormal excitatory cortical neuron activity (Foffani and Obeso, 2018). In addition to motor symptoms caused by dysfunctional striatal activity, excitatory projections to the striatum play a role in executive function (Graybiel, 2016) and thus loss of cortico-striatal glutamatergic synapses may contribute to the development of cognitive symptoms which deserves further exploration. Overall, it is clear that understanding glutamatergic synapses and the interactions between glutamate and dopamine in the striatum, are critical for elucidating the mechanisms responsible for PD symptoms.

## Potential Mechanisms Contributing to Synapse Loss in PD

### Contribution of Axon Arborization and Metabolic Burden on Synapse Loss in PD

Recent reviews describe how axonal morphology potentially contributes to neuron vulnerability in PD (Wong et al., 2019; Gonzalez-Rodriguez et al., 2020). Many neurons harboring Lewy pathology have long, poorly myelinated axons that are highly branched (Del Tredici and Braak, 2016). These neurons include dopamine neurons of the SNc which show extensive arborization with 2–6 × 10^5^, dopamine release sites per axon in the rat (Matsuda et al., 2009), and an estimate of up to 1.5 million release sites in the human brain (Bolam and Pissadaki, 2012). Other neuron subtypes that are vulnerable in PD with extensive arborizations include noradrenergic neurons of the locus coeruleus, serotonergic neurons of the raphe nucleus, and cholinergic neurons of the dorsal motor nucleus of the vagus, pedunculopontine nucleus, and the basal forebrain. The selective vulnerability of these neuron subtypes was recently supported using an injection of fibrils into the pedunculopontine nucleus of the brainstem (Henrich et al., 2020). Although this brain region contains neurons of multiple neurotransmitter phenotypes (GABA and glutamate), only the cholinergic neurons showed fibril-induced formation of α-synuclein inclusions. It has also been shown that expanding axonal arborization of dopamine neurons increases their susceptibility to toxic insults, demonstrating that extensive axon branching contributes to vulnerability (Giguere et al., 2019).

Extensive axonal arborization places a major metabolic burden on these neurons. Dopaminergic SNc neurons have abundant mitochondria and high levels of oxidative phosphorylation to respond to this metabolic demand. Increased oxidative phosphorylation results in elevated levels of damaging reactive oxygen species. Reducing axonal arborization of dopamine neurons decreases levels of oxidative phosphorylation and reactive oxygen species (Pacelli et al., 2015). Oxidation of dopamine may further exacerbate neuron toxicity by promoting abnormal α-synuclein aggregation (Conway et al., 2001; Martinez-Vicente et al., 2008; Burbulla et al., 2017). α-synuclein aggregates themselves increase oxidant stress in dopamine neurons (Dryanovski et al., 2013) and block mitochondrial protein import (Di Maio et al., 2016), thus causing a cascade leading to mitochondrial and synaptic impairments. In addition to oxidant stress, vulnerable neurons with extensive axonal arborization also display oscillations in intracellular Ca^2+^ concentrations. α-synuclein fibrils are cleaved at the C-terminus by the Ca^2+^ dependent protease, calpain which induces soluble α-synuclein to fibrillize. Finally, degradation occurs in acidic lysosomes localized in the neuron soma, and thus retrograde transport of α-synuclein aggregates and damaged mitochondria may be less efficient in neurons with extensive arborization, leading to a toxic build-up in synapses. Overall, these data suggest that neurons with highly branched axons with consequent increased metabolic burden may be particularly at risk and thus be the first to show synaptic toxicity.

### Synapse Loss in PD Caused by α-Synuclein Aggregate Formation

The role α-synuclein plays in early synaptic dysfunction in PD has been previously discussed in a thorough review (Ghiglieri et al., 2018). α-synuclein is a member of a family including α-, ß-, and γ- synuclein. α-synuclein and ß-synuclein localize to the presynaptic terminal. Only α-synuclein contains the non-amyloid component (NAC) domain which is responsible for amyloid formation, and only α-synuclein is found in LBs. In the central nervous system, α-synuclein is expressed in dopamine neurons in the SNc and the ventral tegmental area. However, what is often not appreciated is that it is highly expressed at presynaptic terminals in glutamatergic excitatory neurons, and the role of α-synuclein in excitatory neurons, and the potential contribution to PD warrants further research. Besides, α-synuclein is also highly expressed in cholinergic neurons of the pedunculopontine nucleus and basal forebrain where abnormalities in neuronal function could cause defects in sleep, attention, and cognition. Although α-synuclein shows minimal overlap with GABAergic markers, it is expressed in GABAergic presynaptic terminals of the external plexiform layer of the olfactory bulb, as well as globus pallidus and SNr which likely originate from spiny projection neurons of the striatum, although co-labeling with retrograde tracers would help to prove this (Taguchi et al., 2019; Henrich et al., 2020).

The function of α-synuclein has been difficult to determine; α-synuclein mice are viable with no overt phenotypes. Antisense oligonucleotides to reduce levels of α-synuclein in neurons cause a reduction in synaptic vesicles at the distal pool (Murphy et al., 2000). The first study of α-synuclein knockout mice showed that its absence leads to an increase in striatal dopamine release upon paired stimulation (Abeliovich et al., 2000). α/ß/γ triple knockout mice show enhanced tethering of synaptic vesicles at the active zone (Vargas et al., 2017). More recent studies show that α-synuclein interacts with chaperones and VAMP2 (Chandra et al., 2005; Burré et al., 2010), a member of the SNARE complex involved in synaptic vesicle fusion with the plasma membrane. Deletion of the α-synuclein/VAMP2 binding region prevents clustering of synaptic vesicles. These suggest that α-synuclein clusters synaptic vesicles, acting as a brake on synaptic vesicle release (Scott and Roy, 2012; Wang et al., 2014; Sun et al., 2019). α-synuclein also interacts with DAT (Lee et al., 2001) and abnormal α-synuclein reduces striatal levels of DAT (Giordano et al., 2018), providing another mechanism by which α-synuclein may contribute specifically to the loss of dopamine terminals in the striatum.

Dominantly inherited mutations in α-synuclein and gene and polymorphisms cause PD (Blauwendraat et al., 2020), supporting a role for α-synuclein in the pathogenesis of the disease. LBs are a primary pathologic hallmark of PD (Spillantini et al., 1997). Before the discovery of α-synuclein, LBs were identified using antibodies to ubiquitin or histological stains such as hematoxylin and eosin that only identify rare dense LBs near the nucleus. The discovery that α-synuclein was the major component of Lewy pathology led to the development of highly sensitive antibodies to the protein as well as post-translational modifications of α-synuclein found only in disease brains. These antibodies revealed accumulation of α-synuclein in axons, called Lewy neurites, that are far more abundant in the brain compared to LBs (Duda et al., 2002). Lewy neurites appear before LBs, suggested by pathological staging studies and confirmed with model systems of α-synuclein inclusion formation (Braak et al., 2003; Volpicelli-Daley et al., 2011). Small, phosphorylated α-synuclein aggregates have been observed in presynaptic terminals in the DLB cortex, and in mouse primary neurons with slightly increased levels of α-synuclein (Scott et al., 2010; Colom-Cadena et al., 2017).

**Figure 1:**
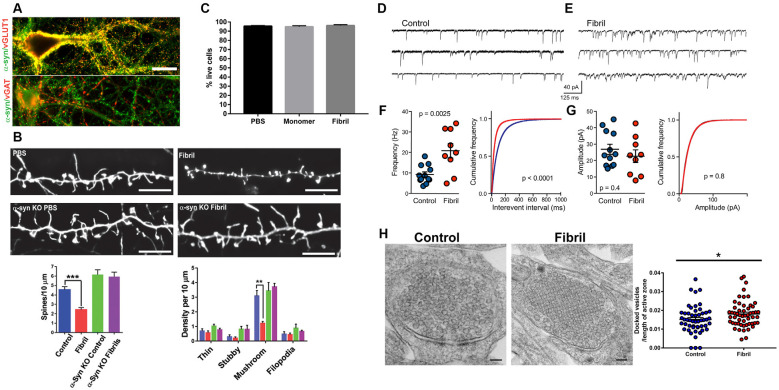
Adapted from Froula et al. (2018). All experiments were performed in primary hippocampal neurons exposed to fibrils (or control) and analyzed 7 days later. **(A)** α-synuclein (green) colocalizes with vGLUT1 (red) at presynaptic terminals in excitatory neurons, but not vGAT (red) at presynaptic terminals in inhibitory neurons. **(B)** Neurons were transfected with LifeAct-GFP. There is a significant reduction in mushroom excitatory spine densities in neurons with α-synuclein inclusions, but not in α-synuclein knockout neurons exposed to fibrils. **(C)** At 7 days following fibril addition to neurons there is no neuron death. **(D–G)** Recording of mEPSCs shows increased mEPSC frequency, but not increased mEPSC amplitudes. **(H)** Electron microscopy of presynaptic terminals shows increased synaptic vesicle docking.*p < 0.05; **p < 0.01; ***p < 0.001.

Excitatory neurons synapse on dendritic spines and presynaptic aggregates of α-synuclein in the cortex of DLB patients correlate with a dramatic reduction in dendritic spines (Kramer and Schulz-Schaeffer, 2007). Reduction of dendritic spines has also been demonstrated in animal models including cortical neurons in mice overexpressing α-synuclein, and in neurons with fibril-induced inclusion formation (Blumenstock et al., 2017; Froula et al., 2018; Wu et al., 2019; Figure 1). Primary hippocampal neurons exposed to α-synuclein fibrils also show reduced spine density at time points well before neuron death, supporting that synapse loss is an early phenotype. Importantly, the addition of fibrils to neurons lacking endogenous α-synuclein does not show dendritic spine loss, indicating synaptic loss occurs in response to the corruption of endogenous α-synuclein.

Whether sequestration of α-synuclein away from its normal localization to the presynaptic terminal into pathologic aggregates results in a loss of function of α-synuclein, some evidence suggests that this may be the case. At early time points following exposure of hippocampal primary neurons to α-synuclein fibrils, there is a significant increase in miniature post-synaptic potential (EPSC) frequency with no change in mEPSC amplitude (Froula et al., 2018; Figure 1). The formation of α-synuclein pathologic aggregates does not cause an increase in synapse number, but causes a decrease in synapse density, as evidenced by the loss of spine density. Therefore, the increase in mEPSCs reflects enhanced exocytosis, similar to the effects of the absence of α-synuclein expression in neurons. These findings require further research, however, it is important to point out that: (1) it is important to study how, in mature synapses, sequestration of normal α-synuclein from the presynaptic terminal into abnormal aggregates contributes to neuronal dysfunction; and (2) studies utilizing models in which α-synuclein (normal or mutant) is overexpressed may not recapitulate what occurs in a PD brain with endogenous (or only slightly higher) levels of α-synuclein.

Postsynaptically, abnormal aggregates of α-synuclein, but not monomeric α-synuclein, disrupt long term potentiation (LTP) in SPNs in the striatum. This is caused by aggregate-induced reduction in cell surface expression of the GluN2A subunit of NMDA receptors, resulting in reduced NMDA receptor current (Tozzi et al., 2016; Durante et al., 2019). Fibril-induced α-synuclein inclusions also cause a major reduction in neuron connectivity, synchronicity, and excitatory tone (Volpicelli-Daley et al., 2011; Froula et al., 2018) which may be caused by reduced cell surface expression of NMDA receptors and/or AMPARs. α-synuclein interacts with spectrin, an actin cross-linking protein, that plays a role in the targeting of NMDA receptors to the cell surface, and abnormal interactions of α-synuclein with spectrin (Ordonez et al., 2018) may be a mechanism by which NMDA receptor cell surface localization is impaired by α-synuclein aggregates.

Early formation of fibril-induced α-synuclein aggregates also results in loss of neuronal connectivity, synchronous firing, and reduced calcium transients (Volpicelli-Daley et al., 2011). Importantly, these defects do not occur in neurons from α-synuclein knockout neurons, demonstrating that the neuronal defects are caused by corruption of endogenous α-synuclein. These perturbations in excitatory neuron function and connectivity may contribute to cognitive dysfunction in PD dementia and DLB.

### Synapse Abnormalities Caused by Leucine-Rich Repeat Kinase 2 (LRRK2) Mutations in PD

**Figure 2:**
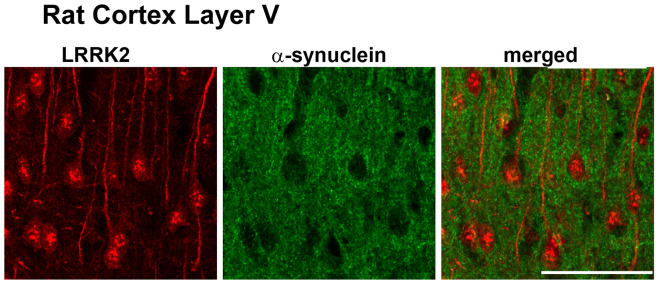
Adapted from West et al. (2014). Leucine-rich repeat kinase 2 (LRRK2) (red) is highly expressed in layer IV/V pyramidal neurons in the mouse motor cortex.

There are outstanding reviews on the role of LRRK2 at the synapse (Nguyen et al., 2019; Pan et al., 2019; Kuhlmann and Milnerwood, 2020; Mancini et al., 2020). Our goal here is to focus on which synapses in the brain LRRK2 may be acting. Mutations in LRRK2 are the most common genetic cause of PD with the G2019S mutation being the most common (Trinh et al., 2014). LRRK2 mutations are autosomal dominant and present similarly to late-onset, sporadic PD. In the brain, LRRK2 is expressed in glutamatergic pyramidal neurons of the cortex (Figure 2), and GABAergic SPNs of the striatum, particularly in the striosome compartment (West et al., 2014). Studies of LRRK2 expression in dopamine neurons of the SNc have produced variable results (Kuhlmann and Milnerwood, 2020): LRRK2 is expressed in dopamine neurons of the SNc in the mouse brain, but is not expressed, or has low levels of expression in the SNc in the rat brain (West et al., 2014). The expression of LRRK2 in the SNc of the human brain has yet to be confirmed. Regardless, confirmed expression in corticostriatal projection neurons and striatal projection neurons of the striatum places LRRK2 in important basal ganglia circuits important for motor function.

Although LRRK2 protein expression in dopamine neurons in the SNc has been difficult to detect, evidence points to a role for LRRK2 in presynaptic dopamine release in the striatum. In bacterial artificial chromosome (BAC) transgenic mice overexpressing wild type LRRK2, dopamine release in the striatum is increased, but in aged BAC transgenic G2019S-LRRK2 mice show reduced striatal levels of dopamine (Li et al., 2010). More recent studies use G2019S-LRRK2 knockin mice because, in transgenic rodent models of mutant LRRK2, expression of LRRK2 can be up to 10-20 fold over endogenous levels. Thus, the knockin mice more faithfully recapitulate the impact of mutant LRRK2 expression in human disease in which the protein is not overexpressed (Volta and Melrose, 2017). Amperometry showed reduced striatal dopamine levels in striatal slices G2019S-LRRK2 knockin mice (Tozzi et al., 2018). Using microdialysis to measure striatal DA levels, 6 month G2019S-LRRK2 knockin mice show normal striatal dopamine release, but 12 months aged G2019S-LRRK2 KI mice showed reduced dopamine levels (Volta et al., 2017), consistent with the findings of the BAC transgenic G2019S-LRRK2 mice (Yue et al., 2015).

Several studies from independent labs confirm a role for LRRK2 in glutamatergic synapses. Cortical cultured neurons from G2019S-LRRK2 mice show substantially increased spontaneous EPSC frequency compared to wild type neurons, without an increase in the number of synapses or a change in mEPSC amplitude (Beccano-Kelly et al., 2014; Plowey et al., 2014; Matikainen-Ankney et al., 2016). Neurons from mice expressing the kinase-dead LRRK2 or neurons treated with LRRK2 kinase inhibitors do not show this increase in glutamatergic activity, demonstrating that increased LRRK2 kinase activity is responsible for the enhanced activity of glutamatergic projections to the striatum. There have been conflicting reports on whether these increased events are action potential-dependent with one report showing insensitivity to tetrodotoxin (Volta et al., 2017) and another showing normalization of sEPSCs activity in G2019S-LRRK2 expressing neurons with (Matikainen-Ankney et al., 2016). The effects on LRRK2 kinase activity on mEPSC frequency could result from increased excitatory synaptic vesicle exocytosis, or altered handling of Ca^2+^ at the presynaptic terminal, particularly mitochondrial Ca^2+^ uptake (Tozzi et al., 2018). A recent study utilizing both R1441C-LRRK2 knockin mice and G2019S-LRRK2 knockin mice showed reduced mEPSC frequencies at both direct and indirect pathway SPNs (Chen et al., 2020). The R1441C LRRK2 mutation increase enhances the postsynaptic activity of protein kinase A (PKA) (Parisiadou et al., 2014; Chen et al., 2020), resulting in increased synaptic targeting of the GluA1 AMPA receptor subunit in direct spiny projection neurons with corresponding increased glutamate uncaging-evoked currents. Future studies examining the impact of mutant LRRK2 on direct and indirect pathway SPN physiology in the context of low striatal dopamine (Graves and Surmeier, 2019) will be of great interest. Overall, these data suggest that research on LRRK2 effects in PD should include excitatory projection neurons which can have a major effect on basal ganglia output.

LRRK2 substrates include proteins involved in the synaptic vesicle endo/exocytosis cycle. An action potential-induced influx of calcium into the presynaptic terminal causes synaptic vesicles to fuse with the plasma membrane to release their neurotransmitters into the synaptic space. Synaptic vesicles are retrieved by different mechanisms including clathrin-mediated endocytosis, activity-dependent bulk endocytosis, or ultrafast endocytosis. In the case of clathrin-mediated vesicle endocytosis, the clathrin coat is shed, the synaptic vesicle is refilled with neurotransmitter and the vesicle can dock and prime for another round of exocytosis (Gan and Watanabe, 2018). LRRK2 acts as a substrate for proteins involved in both synaptic vesicle exocytosis and endocytosis. For example, LRRK2 phosphorylates Rab3a (Steger et al., 2016), a small GTPase that concentrates at the presynaptic terminal that regulates vesicle release probability (Schlüter et al., 2004). LRRK2 also phosphorylates endophilin A1(Arranz et al., 2015) and auxilin (Nguyen and Krainc, 2018), both of which play a role in synaptic vesicle endocytosis by facilitating uncoating of clathrin-coated vesicles (Milosevic et al., 2011). Thus, these data support a role for LRRK2 kinase activity in the synaptic vesicle cycle.

### Synapse Abnormalities Caused by VPS35 Mutations in PD

Similar to LRRK2 mutations, mutations in VPS35 cause an autosomal dominant form of PD with phenotypes similar to late-onset sporadic PD. After LRRK2, VPS35 mutations are the second most common cause of genetic PD. VPS35 associates with VPS26 and VPS29 to form a retromer complex that is involved in membrane transport from the trans-Golgi network to endosomes or the plasma membrane. In polarized neurons, VPS35 plays a role in targeting glutamatergic AMPA receptors to the plasma membrane of dendritic spines (Munsie et al., 2015; Temkin et al., 2017). Knocking down VPS35 impairs long-term potentiation in glutamatergic synapses. Interestingly, expressing a mutant VPS35 associated with Alzheimer’s disease (L625P) rescues the effect of VPS35 knockdown on LTP, whereas expression of the PD-associated mutant (D620N) does not (Temkin et al., 2017), demonstrating that the PD-associated mutant contributes to defects in LTP. The generation of D620N VPS35 knockin mice allowed dissection of the effects of this mutant *in vivo* (Chen et al., 2019). Aged (13 months) D620N VPS35 heterozygous and homozygous mice showed robust axonal damage, identified using gallyas silver, in the striatum, SNc, and hippocampus. Thus, together these studies suggest that PD-associated mutations in VPS35 may cause damage both presynaptically and postsynaptically.

### Synaptic Genes With Mutations That Cause Autosomal Recessive, Early-Onset Parkinsonism

In addition to the dominantly inherited genes that cause late-onset PD with symptoms similar to sporadic PD, there are autosomal recessive gene mutations that cause parkinsonism. These genes cause symptomatic features similar to late-onset, idiopathic PD such as gait disturbances, stiffness, and tremor at rest, as well a loss of dopamine terminals in the putamen and loss of dopamine neurons in the SNc. The autosomal recessive mutations, in general, are early-onset from 20 to 40 years of age in contrast to late-onset PD with an average age of *onset* >60 years of age. Individuals with autosomal recessive gene mutations have slower disease progression and are at lower risk for cognitive decline relative to late-onset PD. Also, autosomal recessive genes can be associated with additional symptoms not typically associated with idiopathic PD such as seizures, pyramidal symptoms, and intellectual disabilities (see below). Further, the brains from these patients generally do not show abundant Lewy pathology (Schneider and Alcalay, 2017). However, it is important to point out that Lewy pathology was assessed in very few patients, at late time points post mortem, with reduced tissue quality, and only focus on LBs although LNs are more abundant and may better correlate with disease. Regardless, several of these autosomal recessive mutations are found in genes that play a mechanistic role in synaptic vesicle release including *PRKN* (parkin), *DNAJC6* (auxilin), and *SYNJ1* (synaptojanin 1) (Nguyen et al., 2019).

Parkin is an E3 ubiquitin ligase, and parkinsonism associated mutations include splice site mutations, deletions, and single base-pair substitutions associated with a loss of function (Dawson and Dawson, 2010). Patients with parkin mutations have an average age of onset before 45 years of age. Symptoms include gait disturbances similar to PD, but unlike PD have a very slow progression, dystonia at the onset of the disease, and are very sensitive to levodopa-induced dyskinesias (Schrag and Schott, 2006; Wickremaratchi et al., 2011). Parkin monoubiquitinates proteins involved in synaptic vesicle endocytosis such as endophilin A1, dynamin, synaptojanin 1 (Cao et al., 2014). Parkin also binds to synaptotagmin-11 which is involved in synaptic vesicle docking (Huynh et al., 2003). Postsynaptically, Parkin ubiquitinates the GluK2 subunit of the kainate receptor (Maraschi et al., 2014) and regulates cell surface expression of AMPA and NMDA receptors (Cortese et al., 2016; Zhu et al., 2018). Parkin knockout mice do not show loss of dopamine neurons in the SNc and have increased levels of extracellular dopamine in the striatum (Goldberg et al., 2003). Microdialysis studies show that aged Parkin knockout rats show reduced evoked release of glycine, an NMDA glutamate receptor co-agonist. Thus, deficiencies in parkin function could affect motor behavior by altering glutamatergic transmission within the striatum (Sassone et al., 2017, 2019; Creed et al., 2019).

Auxilin (DNAJ6) recruits the ATPase, HSC70 to clathrin-coated vesicles to uncoat synaptic vesicles for the repackaging of neurotransmitters (Lemmon, 2001; Yim et al., 2010). Parkinsonism disease-associated mutations in auxilin either cause reduced protein levels or result in the formation of a C-terminal truncation mutant that cannot bind HSC70. In addition to PD-like motor symptoms such as postural instability and slowness of movement, and loss of DAT binding in the striatum, these patients show neurodevelopmental delays, epilepsy, pyramidal signs, and intellectual disabilities (Edvardson et al., 2012; Koroglu et al., 2013; Olgiati et al., 2016; Ng et al., 2020). The absence of auxilin expression in mice causes increased accumulation of clathrin-coated vesicles in primary cortical neurons (Yim et al., 2010). Interestingly, LRRK2 phosphorylates the clathrin binding domain of auxilin, suggesting a convergence of LRRK2 and auxilin on impaired synaptic vesicle endocytosis (Nguyen and Krainc, 2018).

Synaptojanin 1 is a phosphoinositide phosphatase that dephosphorylates PI(4,5)P2 on budding endocytic vesicles initiating recruitment of auxilin and clathrin uncoating (Cremona et al., 1999). Homozygous R258Q and R459P mutations in synaptojanin 1 cause early-onset atypical parkinsonism with PD-like symptoms such as gait disturbances, stiffness, and slowness of movement, along with loss of DAT in the putamen, but unlike PD, show poor responsiveness to levodopa, and cerebral cortex atrophy (Krebs et al., 2013; Quadri et al., 2013; Olgiati et al., 2014; Kirola et al., 2016). Synaptojanin 1 R248Q knock-in mice show abnormally large, dystrophic dopamine terminals in the dorsal striatum (Cao et al., 2017). In one of the few studies to compare different neuron subtypes, it was shown that reduced expression of synaptojanin 1 results in impaired synaptic vesicle endocytosis in dopaminergic midbrain neurons but not cortical neurons, suggesting a possible selective effect on dopaminergic neurons (Pan et al., 2017).

Overall, while these rare juvenile-onset autosomal recessive genes mutations in genes that encode synaptic proteins may not cause PD, they do suggest that dopamine neurons and neurons within basal ganglia circuitry may be particularly susceptible to synaptic defects.

### Role of Risk Genes in Synaptic Defects in PD

Several PD risk genes implicated by Genome-Wide Association Studies (GWAS) also have roles at the synapse including (but not limited to) SH3GL2, SYNJ1, RIMS1, VAMP4, SYT4, ANK2, LRRK2, SNCA, STX1B, SYT11, and VPS35 (Nalls et al., 2014, 2019; Chang et al., 2017; Blauwendraat et al., 2020; Grenn et al., 2020). GWAS and subsequent analyses of gene-phenotypic trait associations (Heilbron et al., 2019) and cell-type-specific expression patterns discovered an enrichment of risk-associated genes in neurons (Bandres-Ciga et al., 2020; Bryois et al., 2020), as well as oligodendrocytes, astrocytes, and endothelium (Reynolds et al., 2019; Bryois et al., 2020). While cell-type-specific variant-associated changes in gene expression remain unexplored due to the technical challenge of performing single-cell transcriptomics and whole-genome sequencing in the human brain, associations between risk variants and gene expression at the tissue level have been found using quantitative trait loci (QTL) mapping (Nica et al., 2010; Hernandez et al., 2012; Westra and Franke, 2014; Guelfi et al., 2020). Variants that affect gene expression in the brain, such as those identified in the SH3GL2 locus, are implicated in synaptic function and endocytic trafficking and have been reviewed elsewhere (Nguyen et al., 2019; Bandres-Ciga et al., 2020).

Considering the roles for α-synuclein in synaptic function in normal and pathological states, investigators often use two-hit models to explore potential synergistic interactions between α-synuclein dysfunction and genes associated with increased risk for PD. However, in light of studies demonstrating roles for PD risk genes in non-neuronal populations (Moehle et al., 2012; Booth et al., 2017; Sliter et al., 2018; Barodia et al., 2019), it is important to consider whether genes of interest are expressed in the same cell types *in vivo* before exploring their potential interactions. With the recent emergence of single-cell RNA sequencing technologies, it is now possible to explore the neuroanatomical colocalization of genes using publicly accessible databases (Saunders et al., 2018; Zeisel et al., 2018; Monzón-Sandoval et al., 2020; Yuste et al., 2020).

**Figure 3:**
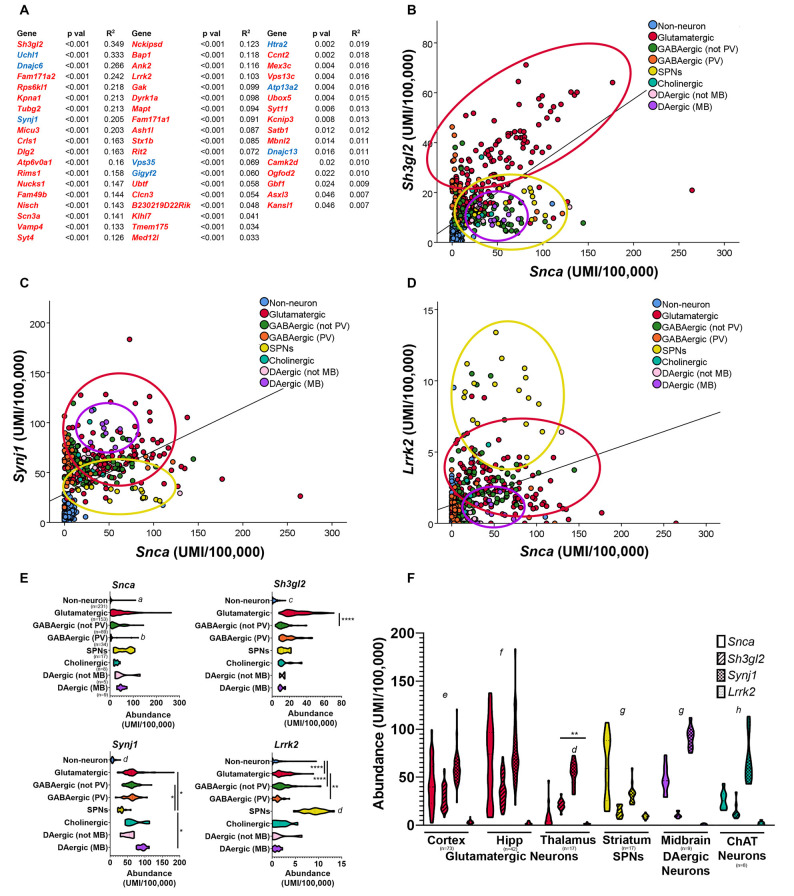
Comparison of neuroanatomical location of *Snca* and other Parkinson’s disease (PD) implicated genes using single-cell transcriptional data in the mouse brain. Correlation analyses of single-cell transcriptional abundance data was performed between *Snca* and genes implicated by PD GWAS; genes with significant correlations to *Snca* across nine regions and > 500 distinct cell subclusters (Saunders et al., 2018) are listed (two-tailed *t*-test; **(A)** GWAS genes in red, *PARK* homologs in blue). Plotting individual subcluster values for *Snca* vs. *Sh3gl2* **(B)** *Synj1* **(C)** and *Lrrk2* **(D)** reveals neuron types which co-express those transcripts. Abundance values for each transcript by cell type are displayed in panel **(E)** and compared across different regions in panel **(F)**. **(E)** Kruskal–Wallis analysis, followed by multiple comparisons. *a*: different than all except GABAergic-PV, *p* < 0.05; *b*: different than all except DAergic–not midbrain and non-neurons, *p* < 0.05; *c*: different than all except dopaminergic–not midbrain, *p* < 0.05, *d*: different than all others, *p* < 0.05. **(F)** two-way ANOVA by region, Tukey’s *post hoc*; *e*: all different from each other, *p* < 0.05; *f*: all different except *Snca* vs. *Synj1*; *g*: all different except *Sh3gl2* vs. *Lrrk2*, *h*: all different except *Snca* vs. *Sh3gl2*; *p* < 0.05. *p < 0.05, **p < 0.01, ****p < 0.0001.

Based on the fact that synucleinopathy is a common pathological characteristic of PD (Courte et al., 2020; Stoyka et al., 2020), we decided to use the single-cell transcriptomic database Dropviz.org (Saunders et al., 2018) to explore the relationship between the cellular localization of *Snca* and GWAS-implicated genes (Figure 3). This database includes gene expression data for 565 transcriptionally distinct cell types across nine brain regions of the mouse, representing one of the most comprehensive, and user-friendly, single-cell databases currently available (Saunders et al., 2018). We compiled a list of mouse homologs of genes implicated by previous PD GWAS (Nalls et al., 2014, 2019; Chang et al., 2017) and genes with *PARK* designations and then performed a correlation analysis of the cell-type-specific distribution of these genes concerning *Snca*. Genes with positive correlations with *p*-values <0.05 are shown in Figure 3A. Gene expression for *Sh3gl2* (endophilin A1) was most highly correlated with *Snca*; graphic demonstration of gene expression by cell subcluster is shown for *Sh3gl2* (Figure 3B). The mRNA for both *Sh3gl2* and *Snca* is enriched in glutamatergic neurons, but not dopaminergic neurons. *Synj1*, a familial gene highly correlated to *Snca* distribution, overlaps with *Snca* in dopaminergic and cholinergic neurons (Figure 3C). In contrast, *Lrrk2* mRNA expression and *Snca* expression overlapped in glutamatergic neurons and SPNs, similar to what has been reported for protein expression (Figure 3D; West et al., 2014), with low expression in dopaminergic neurons (Figure 3E). *Snca*, *Sh3gl2*, *Synj1*, and *Lrrk2* also show differential levels of expression in thalamic glutamatergic neurons, with *Snca* and *Lrrk2* being relatively deficient in thalamic glutamatergic neurons in comparison to cortical glutamatergic neurons (Figure 3F).

An interesting finding that emerges from comparing the neuroanatomical distributions of *Lrrk2* and *Snca* mRNA in the mouse brain is that SPNs express the highest level of *Lrrk2* and as much *Snca* mRNA as glutamatergic neurons of the cortex. LRRK2 protein is selectively localized to the μ-opioid receptor-positive matrix compartment of the striatum (West et al., 2014). A recent physiologic study showed that the PD-associated LRRK2 mutations alter glutamatergic synapse function in direct pathway SPNs (Chen et al., 2020). This draws attention to a relatively under-studied role of LRRK2 and α-synuclein in SPNs and their targets such as GABAergic relay neurons of the SNr. Although LBs are rarely observed in SPNs (Duda et al., 2002), this does not preclude a potential role for α-synuclein in the synapses of SPNs into the SNr; in fact, a reduction in inhibitory neurotransmitter release from SPNs onto the relay neurons of the SNr could disinhibit those neurons, leading to increased inhibitory tone in the thalamus and reduced excitatory drive to the motor cortex. Future experiments could explore whether *LRRK2* or abnormal α-synuclein impair GABA release at the SPN-SNr synapse also in the context of reduced dopamine in the striatum.

It is also important to note that the four genes shown in Figure 3, *Snca*, *Sh3gl2*, *Synj1*, and *Lrrk2*, are enriched in neurons over non-neuronal populations, without preferential enrichment in dopaminergic neurons. This suggests that the vulnerability of these neurons in individuals with disease-associated variation in these genes is not attributable to expression or dysfunction selectively in dopaminergic neurons. However, when genes implicated by PD GWAS studies are considered in aggregate, in the human midbrain and the cortex there is an enrichment of PD risk genes in midbrain dopaminergic neurons, cortical glutamatergic neurons, and oligodendrocytes (Agarwal et al., 2020). These findings are supported by two other recent studies demonstrating the enrichment of PD risk genes in mouse cholinergic neurons and monoaminergic neurons (Nalls et al., 2019; Bryois et al., 2020). Therefore, potential synergism between PD risk gene-associated dysfunction and cell-intrinsic properties of dopaminergic neurons could generate the perfect storm for dopaminergic vulnerability in PD. Future studies are required to determine whether similar scenarios are relevant for other cellular populations with enrichment of PD-associated genes (Day et al., 2006; Graves and Surmeier, 2019).

## Conclusions

Altogether, the evidence discussed above provides strong support for the involvement of synaptic dysfunction in PD etiology. Pathological studies and PET imaging studies in people living with PD support presynaptic abnormalities as an early event in the disease. The development and use of more sensitive and specific ligands such as ^18^F-FE-PE2I could lead to earlier diagnosis of PD potentially improve early PD diagnoses, before intractable neuron loss. The development of PET ligands to SVC2B, which more selectively labels basal ganglia synapses (Janz and Südhof, 1999), may show synapse loss that more specifically distinguishes PD from other neurodegenerative diseases. Also, studies of changes in synaptic markers in the cerebral spinal fluid may help predict cognitive decline in PD (Bereczki et al., 2017).

In the future, we recommend that investigators expand their studies of synaptic function and loss to include non-dopaminergic neuron types including glutamatergic, noradrenergic, and cholinergic neurons and subtypes of inhibitory interneurons, based on the neuroanatomical pattern of candidate gene enrichment. The high co-expression of α-synuclein and endophilin A1 in glutamatergic neurons, as demonstrated here, suggests that glutamatergic neurons may be the cell type of choice to investigate the contributions of endophilin A1 dysfunction to the development of α-synuclein pathology. In contrast, the enrichment of synaptojanin 1 expression within dopamine (Pan et al., 2017) and glutamatergic neurons suggests that some PD-linked mutations may alter synaptic function at multiple synapses, giving rise to more severe phenotypes. Determining the cell- and circuit-specific patterns of gene expression will be important for understanding how alterations in risk gene expression and/or function contribute to specific symptoms and could shed light on mechanisms that contribute to symptoms that are not alleviated by L-DOPA.

## Author Contributions

All authors listed have made a substantial, direct and intellectual contribution to the work, and approved it for publication.

## Conflict of Interest

The authors declare that the research was conducted in the absence of any commercial or financial relationships that could be construed as a potential conflict of interest.
